# Abnormal gene expression in regular and aggregated somatic cell nuclear transfer placentas

**DOI:** 10.1186/s12896-017-0355-4

**Published:** 2017-03-27

**Authors:** Bo-Woong Sim, Chae-Won Park, Myung-Hwa Kang, Kwan-Sik Min

**Affiliations:** 1 Animal Biotechnology, Graduate School of Future Convergence Technology, Institute of Genetic Engineering, Hankyong National University, Ansung, 17579 Korea; 2 National Primate Research Center & Futuristic Animal Resource & Research Center, Korea Research Institute of Bioscience and Biotechnology, Ochang, 28116 Korea; 3 Department of Food and Nutrition, Hoseo University, Asan, 31499 Korea

**Keywords:** Aggregated SCNT, Placenta, Abnormal gene expression

## Abstract

**Background:**

Placental defects in somatic cell nuclear transfer (SCNT) are a major cause of complications during pregnancy. One of the most critical factors for the success of SCNT is the successful epigenetic reprogramming of donor cells. Recently, it was reported that the placental weight in mice cloned with the aggregated SCNT method was significantly reduced. Here, we examine the profile of abnormal gene expression using microarray technology in both regular SCNT and aggregated SCNT placentas as well as in vivo fertilization placentas. One SCNT embryo was aggregated with two 2 to 4 -cell stage tetraploid embryos from B6D2F1 mice (C57BL/6 × DBA/2).

**Results:**

In SCNT placentas, 206 (1.6%) of the 12,816 genes probed were either up-regulated or down-regulated by more than two-fold. However, 52 genes (0.4%) showed differential expression in aggregated SCNT placentas compared to that in controls. In comparison of both types of SCNT placentas with the controls, 33 (92%) out of 36 genes were found to be up-regulated (>2-fold) in SCNT placentas. Among 36 genes, 13 (36%) genes were up-regulated in the aggregated SCNT placentas. Eighty-five genes were down-regulated in SCNT placentas compared with the controls. However, only 9 (about 10.5%) genes were down-regulated in the aggregated SCNT placentas. Of the 34 genes known as imprinted genes, expression was lower in SCNT placentas than that in the controls. Thus, these genes may be the cause of placentomegaly in mice produced post SCNT.

**Conclusions:**

These results suggest that placentomegaly in the SCNT placentas was probably caused by abnormal expression of multiple genes. Taken together, these results suggest that abnormal gene expression in cloned placentas was reduced in a genome-wide manner using the aggregation method with tetraploid embryos.

**Electronic supplementary material:**

The online version of this article (doi:10.1186/s12896-017-0355-4) contains supplementary material, which is available to authorized users.

## Background

Somatic cell nuclear transfer (SCNT) in animals has the potential to be used in a wide range of applications such as species preservation, livestock propagation, and gene targeting [1]. However, this technology is inefficient and results in various abnormalities, leading to high pregnancy losses and neonatal deaths [2]. Although the cloned fetuses attain full term, placentomegaly is a common phenotype observed in cloned animals, irrespective of donor cell type and strain [3–5]. Placentomegaly mainly seems to arise from an abnormally expanded spongiotrophoblast layer with an increased number of glycogen cells, and irregular borderlines between the labyrinthine and spongiotrophoblast layers [6]. Interestingly, the same pattern of placentomegaly was also observed in interspecies hybridization [7, 8], during sperm injection following introduction of somatic cell cytoplasm into an oocyte [9], and in knockout mice with imprinting genes such as *EsxI* [10], *Ipl* [11], and *H19* [12]. Thus, reduction in placental weight is necessary to obtain live and normal fetuses in SCNT.

Several global gene expression analyses using microarrays of more than 10,000 genes were conducted on samples from neonatal placenta to reveal a cluster of abnormally expressed genes [13–15] in the placentas of cloned mice. Of those SCNT-derived embryos that develop to full term, up to 40% have large offspring syndrome (LOS), characterized by hydrops of the fetus, lethargy, and respiratory distress [15–17]. Aggregation of embryonic stem (ES) cells with tetraploid blastocysts has been successfully conducted in mice [18, 19], and chimeric monkeys were produced by the aggregation of 4-cell embryos [20]. We also reported that aggregated SCNT significantly reduced placental weight of cloned mice and improved SCNT efficiency [5]. However, the differences in the genetic pattern of aggregated SCNT embryos and SCNT embryos are not clearly identified. It is therefore very important to analyze the differences in gene expression between the two types of SCNT embryos. In addition, these results will offer important information in solving the problem of lethality in cloned mice production.

In this study, the mRNA expression profiles of SCNT and aggregated SCNT placentas were analyzed using microarray technology. Many genes were found to be differentially expressed between the SCNT and aggregated SCNT placenta. These results further provide evidence supporting the importance of placental abnormalities in cloned animal production.

## Methods

### Placental samples

B6D2F1 mice (C57BL6 × DBA/2) were used to prepare oocytes and cumulus cells. Two-celled embryos were electrofused to produce one-cell tetraploid embryos. Tetraploid embryos were then aggregated with SCNT embryos. One embryo was aggregated with two 2 to 4 cell tetraploid embryos. Detailed methods are described in a previous report [5]. MII oocytes were collected from 6 to 12-week-old females (69 mice). Embryos electrofused were transferred to the foster mothers (47 mice). All recipient females were euthanized at 19.5 dpc and placentas were obtained. Finally, we produced a total of 36 clone mice and placental samples. The protocol was approved by the Committee on Ethics of Animal Experiments at the Hankyong National University (Permit Number: 2014–4).

### Microarray analysis

Total RNA was extracted from five SCNT placentas, six aggregated SCNT placentas, and four controls by using TRIzol reagent (Invitrogen Life Technologies, Carlsbad, USA) and purified using RNeasy columns (Qiagen, Valencia, USA), according to the manufacturers’ protocols.

#### Labeling and purification

Total RNA was amplified and purified using the Ambion Illumina RNA amplification kit (Ambion, Austin, USA) to yield biotinylated cRNA, according to the manufacturer’s instructions. Briefly, 550 ng of total RNA was reverse-transcribed to cDNA using a T7 oligo(dT) primer. Second-strand cDNA was synthesized, transcribed in vitro, and labeled with biotin-NTP.

#### Hybridization and data export

The labeled cRNA samples (0.75 μg) were hybridized to the Illumina MouseRef-8 v2 expression BeadChip (Illumina, Inc., San Diego, USA) for 16–18 h at 58 °C, according to the manufacturer’s instructions. Detection of the array signals was carried out using Amersham Fluorolink Streptavidin-Cy3 (GE Healthcare Bio-Sciences, Little Chalfont, UK), following the bead array manual. Arrays were scanned with an Illumina bead array reader confocal scanner. Array data analysis was performed using Illumina Genome Studio v.2009.2 (Gene Expression Module v.1.5.4).

#### Raw data preparation and statistical analysis

The raw data were extracted using the software provided by the manufacturer (Illumina Genome Studio v.2009.2). The array data were filtered using a detection p-value < 0.05 (a signal value higher than that of the background was required to obtain a detection p-value < 0.05). The selected gene signal value was logarithmically transformed and normalized. Comparative analysis between 2 groups was carried out by p-value evaluation, using the local-pooled-error test (adjusted Benjamini-Hochberg false discovery rate controlled by 5%) and fold-change. Biological ontology-based analysis was performed using the Panther database (http://www.pantherdb.org). In addition to these statistical criteria, genes whose expression differed by more than two-fold were considered differentially expressed between the two groups.

### Quantitative real-time PCR (qRT-PCR)

To validate the microarray data, 12 genes (viz., *Plac1*, *Slc38a4*, *Rprml*, *Pla2g4f*, *Pla2g4d*, *Hsd17β7*, *Hmox1*, *Chac1*, *Car2*, *Slpi*, *Nrn1l*, and *H19*) from different categories were chosen for qRT-PCR analyses. The expression of these genes was either up- and down-regulated by more than two-fold. qRT-PCR was performed with the same placenta used in the microarray analyses.

Primer sequences are outlined in Additional file 1: Table S1 along with the primer annealing temperatures. The primers were designed with the help of Primer 3 software (www.bioneer.co.kr/products/Oligo/CustomOligonucleotides-overview.aspx). Gene expression was analyzed from 5 SCNT placentas and 3 control placentas. The β-actin (ACTB) gene was used as the endogenous control, and the results of the analysis were calculated by using the 2^-ΔΔCT^ method for quantitative relationships.

## Results

### Abnormal gene expression profiles between SCNT, aggregated SCNT, and control placentas

The mouse placentas derived from SCNT, aggregated SCNT, and in vivo fertilized controls were analyzed for their global gene expression patterns using the microarray method. The placental weight of the control, aggregated SCNT, and SCNT was 0.147 g (*n* = 8), 0.215 g (*n* = 9), and 0.287 (*n* = 27), respectively, as previously described [5].

**Fig. 1:**
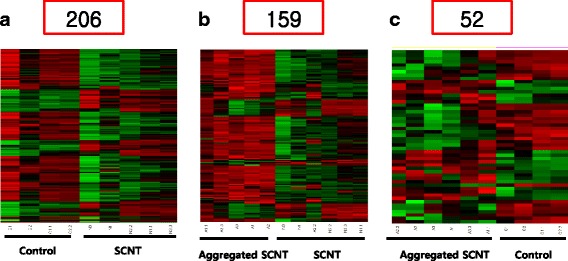
Hierarchical cluster of expression profiles in placentas of both types of cloned mice and the in vivo fertilization placenta (control). **a** A cluster of 206 genes between the control and the SCNT placentas, (**b**) a cluster of 159 genes between aggregated SCNT and SCNT placentas, and (**c**) a cluster of 52 genes between aggregated SCNT and control placentas. Expression of more than 2-fold difference is indicated by increasing red intensity and green indicates reduced expression. SCNT, con = control

Gene transcription levels were detected in the microarray analysis using 12,816 gene probes. Genes showing >2-fold difference in expression were identified for 15 placentas (SCNT, 5; aggregation SCNT, 6; control 4). Figure 1a shows the gene expression differences between control and SCNT placentas. The expression of 206 (1.6%) of 12,816 genes was found to differ by at least two-fold between the SCNT placentas and the controls. Similarly, 159 genes showed different expression between the SCNT placentas and the aggregated SCNT placentas (Fig. 1b). However, 52 (0.4%) genes showed difference (>2-fold) in expression between the aggregated SCNT placentas and the controls (Fig. 1c).

### Co-up- and down-regulated genes in SCNT and aggregated SCNT placentas compared with controls

Many of the differentially expressed gene probes were common to the two types of clones, whereas some were deregulated either in SCNT placentas or in the aggregated SCNT placentas alone. Of the 36 genes up-regulated in SCNT placentas, 10 (27.7%) were commonly up-regulated in the aggregated SCNT placenta group. Ten genes [*Pla2g4f*, *Car2*, *Tekt1* (probe1), *Tekt1* (probe2), *Pla2g4d*, *Rprm1*, *Hsd17b7*, *Hic1*, *Hmox1*, and *Coll15a1*] were up-regulated (>2-fold) in both SCNT and aggregated SCNT placentas (Table 1). However, three genes (*Abcc10*, *Prss22*, and *Slc22a18*) were only up-regulated in the aggregated SCNT placentas. The number of genes showing >2-fold up-regulation in the SCNT placentas was decreased by 33% (from 39 to 13) in the placentas obtained by aggregated SCNT.

**Table 1:** Genes that were up-regulated (fold change) in SCNT placentas versus in control placentas

Gene symbol	Accession No.	Folder Δ SCNT/Con	Folder Δ Agg/Con
Significantly elevated in NT alone			
Tiam1	NM_009384.2	2.32^*^	1.91
Dao1	NM_010018.2	2.36^*^	1.88
Mmp15	NM_008609.3	2.27^*^	1.78
Cxcl1	NM_008176.1	2.42^*^	1.84
Aldh1a3	NM_053080.2	2.08^*^	1.54
E130203B14Rik	NM_178791.4	2.05^**^	1.50
Cdx2	NM_007673.3	2.74^*^	1.98
Plac1	NM_019538.3	2.73^**^	1.86
Slc38a4	NM_027052.3	2.32^**^	1.55
Irs3	NM_010571.3	2.23^*^	1.46
Ldoc1	NM_001018087.1	2.78^*^	1.78
Gna14	NM_008137.3	3.05^**^	1.87
Galk1	NM_016905.2	2.32^*^	1.40
Serpinb9d	NM_011460.1	2.05^*^	1.23
Prl7c1	NM_026206.2	3.22^**^	1.89
Prl2c1	NM_001045532.1	2.40^**^	1.36
Ms4a10	NM_023529.2	2.64^*^	1.49
Sbsn	NM_172205.3	3.07^**^	1.64
Prl2c5	NM_181852.1	2.51^*^	1.33
Sbsn	NM_172205.3	3.32^*^	1.63
Serpinb9d	NM_011460.1	3.03^**^	1.46
Prl4a1	NM_011165.3	2.69^**^	1.26
Ghrh	NM_010285.2	2.15^*^	−1.06
Ada (probe 1)	NM_007398.3	2.86^**^	1.54
Ada (probe 2)	NM_007398.3	3.11^**^	1.92
Ermap	NM_013848.1	2.84^*^	1.12
Col15a1	NM_009928.3	2.70^*^	2.59
Hmox1	NM_010442.1	2.73^**^	2.35
Rprml	NM_001033212.1	3.25^**^	2.69
Hsd17b7	NM_010476.3	3.24^**^	2.45
Hic1	NM_010430.2	3.02^*^	2.25
Pla2g4f	NM_001024145.1	4.10^**^	2.69
Pla2g4d	NM_001024137.1	3.70^**^	2.32
Tekt1 (probe 1)	NM_011569.2	3.40^*^	2.05
Tekt1 (probe 2)	NM_011569.2	3.90^*^	2.26
Car2	NM_009801.3	3.95^*^	2.09
Significantly elevated in aggregation			
Abcc10	NM_170680.2	1.01	2.96^*^
Prss22	NM_133731.1	−1.11	2.44^*^
Slc22a18	NM_008767.2	1.32	2.95^*^

*SCNT* somatic cell nuclear transfer

*Significantly elevated in NT alone

*p < 0.05; **p < 0.01

In Table 2, we have identified the genes showing significant downregulation (>2-fold) in the SCNT, aggregated SCNT, and control placentas. Eighty-five genes were found to be downregulated in SCNT placentas in comparison with their expression in the controls. In contrast, 16 of these genes (18%) *(Sftpd, Tph, 2010109103Rik, Cbx7, Osta, Serpina10, Macb, Fcgrt, Bex2, 84304C8G22Rik, Acox2, Vdr, Dab2, Cfi, Ltf, and Dab2*) were upregulated in aggregated SCNT placentas in comparison with their expression in the controls. Five of the 85 genes (6%) (*Inhba*, *Chac1*, *Nrn11*, *Tnfrsf11b*, and *Slpi*) were down-regulated (>2-fold) in both types of SCNT placentas compared to their expression in the controls (Table 2). Four genes (*Nppb*, *1200015F23Rik*, *Uap1*, and *Ctsm*) were only downregulated (>2-fold) in the aggregated SCNT placentas. These abnormalities in gene expression were significantly reduced with the use of the aggregated SCNT method.

**Table 2:** Genes that were down-regulated (fold change) in SCNT placentas versus in control placentas

Gene symbol	Accession No.	Folder Δ SCNT/Con	Folder Δ Agg/Con	Gene symbol	Accession No.	Folder Δ SCNT/Con	Folder Δ Agg/Con
1300017J02Rik	NM_027918.1	−2.67^**^	−1.34	Cbx7	NM_144811.3	−3.10^**^	1.12
Ly6g6c	NM_023463.3	−3.82^**^	−1.92	Osta	NM_145932.3	−2.88^*^	1.18
Pcyox1	NM_025823	−2.16^**^	−1.10	Entpd2	NM_009849.1	−3.64^**^	−1.10
Lamb3	NM_008484.2	−2.94^*^	−1.53	Bex4	NM_212457.1	−3.50^**^	−1.07
Ang	NM_007447.2	−2.26^*^	−1.20	Serpina10	NM_144834.3	−2.39^*^	1.36
Bmp4	NM_007554.2	−2.59^**^	−1.42	Fga	NM_010196.1	−3.51^**^	−1.10
LOC100046120	XM_001475611.1	−2.62^**^	−1.51	1700045I19Rik	NM_028842.1	−3.47^**^	−1.11
Aqp8	NM_007474.1	−2.32^**^	−1.36	Fga	NM_010196.2	−3.47^**^	−1.12
Heph	NM_010417.1	−2.52^*^	−1.51	BC040758	NM_001033364.1	−3.34^**^	−1.12
Serping1	NM_009776.1	−2.35^**^	−1.43	Maob	NM_172778.1	−2.95^*^	1.01
Doxl2	NM_001029987.1	−2.18^**^	−1.33	BC040758	NM_001033364.1	−3.34^**^	−1.12
Saa3	NM_011315.3	−2.95^**^	−1.79	Fcgrt	NM_010189.1	−2.75^**^	1.07
Aqp8	NM_007474.1	−2.19^**^	−1.34	Gdpd3	NM_024228.2	−3.60^**^	−1.23
Klk4	NM_019928.1	−2.79^**^	−1.72	Cldn2	NM_016675.3	−2.93^**^	−1.01
Serpind1	NM_008223.2	−2.67^*^	−1.67	Psca	NM_028216.1	−4.43^**^	−1.53
Abp1	NM_029638.1	−2.06^**^	−1.33	1600015I10Rik	NM_001081273.1	−4.64^**^	−1.61
Krt14	NM_016958.1	−2.11^*^	−1.42	Bex2	XM_977338.1	−2.52^*^	1.14
Scg5	NM_009162.3	−2.77^*^	−1.95	Apom	NM_018816.1	−3.27^**^	−1.16
Mustn1	NM_181390.2	−2.05^*^	−1.65	8430408G22Rik	NM_145980.1	−2.12^*^	1.31
Tacstd2	NM_020047.3	−2.07^**^	−1.71	Acox2	NM_053115.1	−2.53^**^	1.08
Sfrp5	NM_018780.2	−2.02^**^	−1.88	Spp2	NM_029269.1	−2.91^**^	−1.06
Sftpd	NM_009160.1	−4.14^**^	1.13	Vdr	NM_009504.3	−2.19^*^	1.21
Tph1	NM_009414.2	−2.65^*^	1.59	Itih3	NM_008407.1	−2.64^*^	−1.00
2010109I03Rik	NM_025929.2	−2.75^**^	1.47	Spink3	NM_009258.2	−2.81^*^	−1.08
Amn	NM_033603.2	−4.03^**^	−1.14	Apoa2	NM_013474.1	−2.77^*^	−1.07
Gene symbol	Accession No.	Folder Δ SCNT/Con	Folder Δ Agg/Con	Gene symbol	Accession No.	Folder Δ SCNT/Con^*^	Folder Δ Agg/Con
Significantly reduced in NT				Significantly reduced in NT			
Tfrc	NM_011638.3	−3.08^**^	−1.20	C3	NM_009778.1	−2.23^*^	−1.06
Kng1	NM_023125.2	−3.07^*^	−1.20	Igfbp6	NM_008344.2	−3.64^**^	−1.76
Fcgr3	NM_010188.4	−2.56^*^	−1.02	Popdc3	NM_024286.1	−3.68^**^	−1.79
Dab2	NM_001008702.1	−2.34^**^	1.06	Muc13	NM_010739.1	−2.09^**^	−1.03
Gldc	NM_138595.1	−2.66^**^	−1.08	Lcn2	NM_008491.1	−2.42^*^	−1.20
Serpina1b	NM_009244.4	−2.80^**^	−1.14	Inhba	NM_008380.1	−2.81^**^	−2.25
Tfrc	NM_011638.3	−2.87^**^	−1.18	Chac1	NM_026929.3	−2.27^*^	−2.09
Cfi	NM_007686.2	−2.20^*^	1.10	Nrn1l	NM_175024.3	−2.49^*^	−2.49^*^
Ltf	NM_008522.3	−2.30^**^	1.05	Tnfrsf11b	NM_008764.3	−2.15^*^	−2.30
Gpc3	NM_016697.2	−2.50^*^	−1.05	Slpi	NM_011414.2	−4.56^**^	−2.09
Mgst1	NM_019946.3	−2.51^*^	−1.06				
Fgg	NM_133862.1	−2.95^**^	−1.25	Significantly reduced in aggregation			
Nr1h4	NM_009108.1	−3.16^*^	−1.35	Nppb	NM_008726.3	−1.46	−3.14^**^
Dab2	NM_023118.1	−2.31	1.01	1200015F23Rik	NM_001033136.2	−1.00	−2.71^*^
Kng1	NM_023125.2	−2.87	−1.24	Uap1	NM_133806.4	1.27	−2.72^**^
Slc7a9(prob1)	NM_021291.1	−3.04	−1.34	Ctsm	NM_022326.3	1.12	−4.82^**^
Slc7a9(prob2)	NM_021291.1	−2.82	−1.24				
Irf6	NM_016851.2	−2.43	−1.08				
Trf	NM_133977.2	−2.37	−1.08				
Serpina1d	NM_009246.3	−2.59	−1.19				
Sema4a	NM_013658.2	−2.60	−1.20				
Serpina1b	NM_009244.4	−2.62	−1.21				
Gipc2	NM_016867.1	−2.56	−1.19				
Kng2	NM_201375.1	−2.59	−1.20				
Slc27a2	NM_011978.2	−2.96	−1.40				

*Significantly reduced in NT alone, *p < 0.05; **p < 0.01

### Up-and down-regulated genes in SCNT and aggregated SCNT placentas

We next compared the expression patterns of the up-regulated (>2-fold) genes between SCNT and aggregated SCNT placentas. Twenty-one genes showed higher expression in SCNT placentas than in aggregated SCNT placentas. Four of these genes (19%; *Ermap*, *Prl4a1*, *Sbsn*, and *Serpinb9d*) also showed >2-fold higher expression in the SCNT placentas than in the controls. Additional seven genes (30%; *Atf4*, *Atp6v1d*, *Fmr1nb*, *Gnaq*, *Riok1*, *Tomm22*, and *Zfp330*) showed >2-fold lower expression in the aggregated SCNT placentas than in the controls (Table 3). We also analyzed the expression patterns of the down-regulated (*p*<0.05 and >2-fold) genes between both SCNT groups. Of the 102 genes that showed lower expression in the SCNT placentas than in the aggregated SCNT group, 53 (51%) had even lower expression than that observed in the controls. However, four genes were up-regulated and one gene (*Slpi*) was down-regulated in the aggregated SCNT placentas compared with that in the controls (Table 4).

**Table 3:** Comparison of the genes that were up-regulated (fold change) in SCNT placentas versus in aggregated SCNT placentas

Gene symbol	Accession No.	Folder Δ SCNT/Agg	Folder Δ SCNT/Con	Folder Δ Agg/Con
Cmas	NM_009908.1	3.15^**^	1.85^**^	−1.70
Ermap	NM_013848.1	2.53^**^	2.84^*^	1.12
Prl2a1	NM_019991.1	2.22^**^	1.30^*^	−1.70
Prl4a1	NM_011165.3	2.13^**^	2.69^**^	1.26
Sbsn	NM_172205.3	2.03^*^	3.32^**^	1.63
Serpinb9d	NM_011460.1	2.08^**^	3.03^**^	1.46
2310039H08Rik	NM_025966.3	2.11^*^	1.28	−1.65
Gpn2	NM_133884.1	2.14^*^	1.14	−1.88
H2-Q5	NM_010393.1	2.10^**^	1.45	−1.45
Matn1	NM_010769.1	2.30^*^	1.81	−1.27
Mlycd	NM_019966.2	2.08^**^	1.19	−1.74
Pacsin1	NM_011861.2	2.20^*^	1.28	−1.71
Prcp	NM_028243.2	2.12^**^	1.37	−1.55
Stab2	NM_138673.2	2.03^*^	1.32	−1.54
Atf4	NM_009716.2	2.42^*^	1.02	−2.36
Atp6v1d	NM_023721.2	2.43^**^	−1.01	−2.46
Fmr1nb	NM_174993.1	2.35^**^	1.14	−2.07
Gnaq	NM_008139.5	2.75^**^	1.23	−2.25
Riok1	NM_024242.2	2.37^**^	1.08	−2.19
Tomm22	NM_172609.3	2.35^**^	1.04	−2.26
Zfp330	NM_145600.1	2.58^**^	1.14	−2.26

Significantly elevated in SCNT

*p < 0.05; **p < 0.01

**Table 4:** Comparison of the genes that were down-regulated (fold change) in SCNT placentas versus in aggregated SCNT placentas

Gene symbol	Accession No.	Folder Δ SCNT/Agg	Folder Δ SCNT/Con	Folder Δ Agg/Con
C6	NM_016704.1	−2.05^**^	−1.75^*^	1.17
5033414D02Rik	NM_026362.1	−2.04^**^	−1.86^*^	1.10
Sftpd	NM_009160.1	−4.68^**^	−4.14^**^	1.13
Tph1	NM_009414.2	−4.20^**^	−2.65^*^	1.59
2010109I03Rik	NM_025929.2	−4.04^**^	−2.75^**^	1.47
Amn	NM_033603.2	−3.55^**^	−4.03^**^	−1.14
Cbx7	NM_144811.3	−3.46^**^	−3.10^**^	1.12
Osta	NM_145932.3	−3.40^**^	−2.88^*^	1.18
Entpd2	NM_009849.1	−3.30^**^	−3.64^**^	−1.10
Bex4	NM_212457.1	−3.26^**^	−3.50^**^	−1.07
Serpina10	NM_144834.3	−3.25^**^	−2.39^*^	1.36
Fga	NM_010196.1	−3.18^**^	−3.51^**^	−1.10
1700045I19Rik	NM_028842.1	−3.13^**^	−3.47^**^	−1.11
Fga	NM_010196.2	−3.10^**^	−3.47^**^	−1.12
BC040758	NM_001033364.1	−2.99^**^	−3.34^**^	−1.12
Maob	NM_172778.1	−2.99^*^	−2.95^*^	1.01
BC040758	NM_001033364.1	−2.97^**^	−3.34^*^	−1.12
Fcgrt	NM_010189.1	−2.94^**^	−2.75^**^	1.07
Cldn2	NM_016675.3	−2.91^**^	−2.93^**^	−1.01
Psca	NM_028216.1	−2.90^**^	−4.43^**^	−1.53
1600015I10Rik	NM_001081273.1	−2.89^**^	−4.64^**^	−1.61
Bex2	XM_977338.1	−2.88^**^	−2.52^*^	1.14
Apom	NM_018816.1	−2.81^**^	−3.27^**^	−1.16
8430408G22Rik	NM_145980.1	−2.77^**^	−2.12^*^	1.31
Acox2	NM_053115.1	−2.74^**^	−2.53^**^	1.08
Spp2	NM_029269.1	−2.74^**^	−2.91^**^	−1.06
Muc13	NM_010739.1	−2.03^*^	−2.09^**^	−1.03
Lcn2	NM_008491.1	−2.01^*^	−2.42^**^	−1.20
Slpi	NM_011414.2	−2.18^**^	−4.56^**^	−2.09
Aig1	NM_025446.1	−2.81^**^	−1.09	2.57
Wfdc2	NM_026323.2	−2.02^*^	−1.01	2.00
Snca	NM_009221.2	−2.86^**^	−1.56	1.84
Eraf	NM_133245.1	−2.84^**^	−1.61	1.77
Snca	NM_009221.2	−2.66^**^	−1.42	1.87
Slco2b1	NM_175316.3	−2.64^**^	−1.88	1.41
Hpx	NM_017371.1	−2.52^*^	−1.70	1.49
1810007E14Rik	NM_025308.1	−2.52^**^	−1.66	1.52
Slc4a1	NM_011403.1	−2.49^**^	−1.48	1.68
Alas2	NM_009653.1	−2.46^**^	−1.41	1.75
Ctsh	NM_007801.1	−2.43^**^	−1.97	1.24
Ttr	NM_013697.3	−2.34^*^	−1.65	1.42
Dnmt3l	NM_019448.2	−2.31^**^	−1.36	1.69
Abhd14b	NM_029631.2	−2.25^*^	−1.77	1.27
Cish	NM_009895.3	−2.22^*^	−1.99	1.12
Vdr	NM_009504.2	−2.22^**^	−1.89	1.17
Dnmt3l	NM_001081695.1	−2.21^**^	−1.40	1.58
Gstm1	NM_010358.4	−2.19^*^	−1.67	1.31
Magi1	NM_001029850.2	−2.18^**^	−1.21	1.80
Upk3b	NM_175309.3	−2.16^*^	−1.70	1.27
Clic6	NM_172469.3	−2.16^*^	−1.73	1.24
LOC100044204	XM_001471696.1	−2.14^**^	−1.88	1.14
Igfbp2	NM_008342.2	−2.12^**^	−1.65	1.29
Vdr	NM_009504.3	−2.65^**^	−2.19^*^	1.21
Itih3	NM_008407.1	−2.63^**^	−2.64^*^	−1.00
Apoa2	NM_013474.1	−2.59^**^	−2.77^*^	−1.07
Tfrc	NM_011638.3	−2.56^**^	−3.08^**^	−1.20
Kng1	NM_023125.2	−2.56^**^	−3.07^*^	−1.20
Fcgr3	NM_010188.4	−2.52^**^	−2.56^*^	−1.02
Dab2	NM_001008702.1	−2.48^**^	−2.34^*^	1.06
Gldc	NM_138595.1	−2.47^*^	−2.66^**^	−1.08
Serpina1b	NM_009244.4	−2.45^**^	−2.80^**^	−1.14
Tfrc	NM_011638.3	−2.44^**^	−2.87^**^	−1.18
Cfi	NM_007686.2	−2.43^**^	−2.20^*^	1.10
Ltf	NM_008522.3	−2.40^**^	−2.30^**^	1.05
Gpc3	NM_016697.2	−2.38^**^	−2.50^*^	−1.05
Mgst1	NM_019946.3	−2.37^**^	−2.51^*^	−1.06
Fgg	NM_133862.1	−2.36^**^	−2.95^**^	−1.25
Nr1h4	NM_009108.1	−2.33^**^	−3.16^*^	−1.35
Kng1	NM_023125.2	−2.32^**^	−2.87^**^	−1.24
Slc7a9	NM_021291.1	−2.28^*^	−3.04^*^	−1.34
Slc7a9	NM_021291.1	−2.27^**^	−2.82^*^	−1.24
Irf6	NM_016851.2	−2.25^**^	−2.43^*^	−1.08
Trf	NM_133977.2	−2.19^**^	−2.37^**^	−1.08
Sema4a	NM_013658.2	−2.16^*^	−2.60^*^	−1.20
Serpina1b	NM_009244.4	−2.16^**^	−2.62^**^	−1.21
Gipc2	NM_016867.1	−2.15^**^	−2.56^*^	−1.19
Kng2	NM_201375.1	−2.15^**^	−2.59^*^	−1.20
Igfbp6	NM_008344.2	−2.06^**^	−3.64^*^	−1.76
Ostb	NM_178933.2	−2.08^**^	−1.83	1.14
Pmp22	NM_008885.2	−2.06^*^	−1.98	1.04
Fbp2	NM_007994.3	−3.62^**^	−2.27	1.59
Apoa4	NM_007468.2	−3.25^**^	−3.06	1.06
Bex2	NM_009749.1	−3.19^**^	−2.71	1.18
Eps8l3	NM_133867.1	−3.00^**^	−2.13	1.40
Slc23a3	NM_194333.3	−2.92^**^	−2.97	−1.02
Lbp	NM_008489.2	−2.89^**^	−2.39	1.21
Pdzk1ip1	NM_026018.2	−2.81^**^	−2.62	1.07
Upk3b	NM_175309.3	−2.78^**^	−2.27	1.23
Sepp1	NM_009155.3	−2.74^**^	−2.60	1.06
Cyp2c70	NM_145499.1	−2.65^**^	−2.28	1.16
Fmo1	NM_010231.2	−2.59^**^	−2.44	1.06
Upk3b	NM_175309.3	−2.43^**^	−2.05	1.19
Rnase4	NM_201239	−2.32^**^	−2.76	−1.19
Apoc1	NM_007469.3	−2.31^*^	−2.07	1.11
Apoc1	NM_007469.3	−2.30^*^	−2.07	1.11
Gpc3	NM_016697.2	−2.25^*^	−2.23	1.01
Timd2	NM_134249.3	−2.16^*^	−2.39	−1.11
Pcdh24	NM_001033364.2	−2.15^**^	−2.29	−1.07
Gldc	NM_138595.1	−2.12^**^	−2.18	−1.03
Bglap-rs1	NM_031368.3	−2.03^*^	−2.13	−1.05
Abcc10	NM_170680.2	−2.93^**^	1.01	2.96^*^
Prss22	NM_133731.1	−2.70^**^	−1.11	2.44^*^

Significantly reduced in SCNT

*p < 0.05; **p < 0.01

### Biological process and functional prediction of the differentially expressed genes

We further analyzed the data to obtain more insights into the biological processes and functions of the differentially expressed genes. The distribution of 206 genes that showed differential expression (of at least 2-fold) between the SCNT placentas and the controls, as well as their distribution in different gene ontology (GO) categories, is given in Additional file 2: Figure S1 and Additional file 3: Figure S2. GO-based analysis was performed using the Panther database (http://www.pantherdb.org). The GO terms under the category “biological process” that were most represented (>7%) in the SCNT placentas included “signal transduction (14%),” “immunity and defense (10%),” “transport (8%),” “protein metabolism and modification (8%),” and “developmental processes (7%).” In particular, the proportion of genes under “biological process unclassified” was 12% (Additional file 2: Figure S1). Under the category of “molecular function,” genes were classified into 27 categories by GO, the most represented ones being those for “select-regulatory molecule (9%),” “signaling molecule (9%),” “transporter (7%),” and “oxidoreductase (7%).” Sixteen percent of genes were categorized under “molecular function unclassified” (Additional file 3: Figure S2). The number of classified genes constitutes the number of categories calculated after excluding the overlapping ones.

### Imprinting gene expression in the clones

Next, we focused on the expression profiles of imprinted genes in placentas. Of the 34 imprinted genes identified, none showed differences (>2-fold) in expression between the SCNT and aggregated SCNT placentas. Of these 34 genes, two *Slc22a18; Slc38a4* showed higher expression (>2-fold) in the aggregated SCNT placenta than in the controls. Conversely, *Igfbp6* was down-regulated (>2-fold) in the SCNT placentas than in the controls. Six genes *Ppp1r9a*, *Tssc4*, *Ascl2*, *Cd81*, *Pon2*, and *Slc22a2* were placenta-specific imprinted genes that are expressed on the maternal allele in mice and humans. All these six genes were similarly expressed between the SCNT, aggregated SCNT, and control placentas. Most of the imprinted genes showed lower expression in SCNT placentas than in the controls (Table 5).

**Table 5:** Imprinted gene expression

Gene symbol	Accession No.	Folder Δ SCNT/Con	Folder Δ Agg/Con	Gene symbol	Accession No.	Folder Δ SCNT/Con^*^	Folder Δ Agg/Con
Atp10a	NM_009728.1	1.22	1.25	Slc22a4	NM_019687.3	−1.11	1.35
Cdkn1c	NM_009876.3	1.01	−1.02	Ube3a	NM_011668.2	−1.05	−1.03
Dcn	NM_007833.4	−1.56	−1.18	Zim1	NM_011769.3	−1.32	−1.27
Gnas	NM_201617.1	−1.10	1.04	Slc38a4	NM_027052.3	2.32^**^	1.55
Meg1/Grb10	NM_010345	−1.75	−1.05	Cdkn1c	NM_009876.3	1.01	−1.02
H19	NR_001592.1	1.02	1.10	Igfbp6	NM_008344.2	−3.64^**^	−1.76
Igf2	NM_010514.2	−1.01	−1.02	Igfbp2	NM_008342.2	−1.65	1.29
Igf2r	NM_010515.1	−1.35	−1.11	Xist	NR_001463.2	1.24	1.03
Impact	NM_008378.2	−1.20	1.04	Ppp1r9a^a^	NM_181595.3	−1.44	−1.05
Ins1	NM_008386.3	−1.03	−1.01	Tssc4^a^	NM_020285.1	1.06	−1.02
Ins2	NM_008387.3	1.26	1.10	Ascl2^a^	NM_008554.2	1.81	1.52
Peg1/Mest	NM_008590.1	−1.41	1.00	Cd81^a^	NM_133655.1	−1.42	−1.18
Peg10	NM_001040611.1	1.10	1.07	Pon2^a^	NM_183308.2	1.20	1.16
Peg3	NM_008817.2	−1.16	1.04	Slc22a2^a^	NM_013667.2	−1.02	1.06
Rasgrf1	NM_011245.1	1.17	1.24				
Ndn	NM_010882.3	−1.23	−1.06				
Nnat	NM_010923.2	−1.62	1.13				
Slc22a18	NM_008767.2	1.32	2.95^*^				

Significantly reduced in SCNT

^a^Placenta-specific imprinted genes in mice and human

*p < 0.05; **p < 0.01

### Gene expression analysis by qRT- PCR

**Fig. 2:**
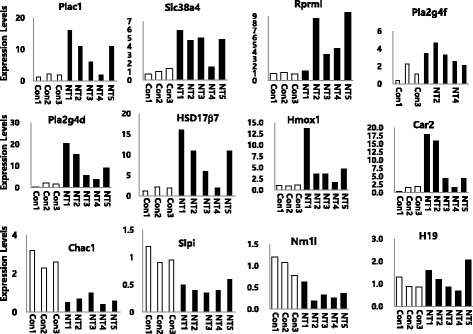
Quantitative real time PCR analysis. Twelve genes from different categories were chosen for qRT-PCR analyses. Eight genes were upregulated in the SCNT placentas. The other three genes were upregulated in the control group. H19 showed almost the same expression pattern. The β-actin gene was used as the endogenous control

To validate our microarray analysis, we performed qRT-PCR analysis for the 12 genes identified (Fig. 2). With regard to the down-regulated genes that were identified by microarray in SCNT placentas, three *Chac1*, *Slpi*, and *Nrn1l* were confirmed to be down-regulated in the SCNT placentas by qRT-PCR. Eight genes *Plac1*, *Slc38a4*, *Rprml*, *Pla2g4f*, *Pla2g4d*, *Hsd17β7*, *Hmox1*, and *Car2* were identified as up-regulated by >2-fold in the SCNT placentas. Six of these genes *Slc38a4*, *Rprml*, *Pla2g4f*, *Pla2g4d*, *Hsd17b7,* and *Car2* were confirmed to be up-regulated by qRT-PCR (Table 6). The fold change in expression of *H19*, a known imprinting gene, was found to be 1.1 in the microarray analysis indicating, that its expression level was similar in the SCNT placentas and controls, and confirming that the results of qRT-PCR correlated with those of the microarray analysis. However, quantitative gene expression analysis in individual placentas showed that the expression levels varied over a wide range among the five SCNT placentas from the normal placental state.

**Table 6:** Validation of microarray results using quantitative real-time PCR

Gene symbol	Accession No.	Microarray folder Δ, SCNT/Con	Expression	qRT-PCR folder Δ, SCNT/Con
Plac1	NM_019538.3	2.73^**^	↑NT	6.54
Slc38a4	NM_027052.3	2.32^**^	↑NT	4.04^*^
Rprml	NM_001033212.1	3.25^**^	↑NT	4.51
Pla2g4f	NM_001024145.1	4.10^**^	↑NT	3.13^*^
Pla2g4d	NM_001024137.1	3.70^**^	↑NT	9.04
Hsd17b7	NM_010476.3	3.24^**^	↑NT	7.66
Hmox1	NM_010442.1	2.73^**^	↑NT	4.30
Chac1	NM_026929.3	−2.27^*^	↓NT	−8.41
Car2	NM_009801.3	3.95^**^	↑NT	6.11
Slpi	NM_011414.2	−4.56^**^	↓NT	−4.06
Nrn1l	NM_175024.3	−2.49^**^	↓NT	−3.05^**^
H19	NR_001592.1	1.10		1.19

*Fold change values with superscripts were significantly different

*p < 0.05; **p < 0.01

## Discussion

This study was undertaken to profile gene expression in the placentas of SCNT and aggregated SCNT and in vivo fertilization placentas by microarray analysis. We obtained a list of up- and down-regulated genes showing >2-fold difference between the SCNT and control placentas. The number of upregulated genes was lower by 75% in the aggregated SCNT placentas than in the SCNT placentas (206 → 52). The gene expression profiles of aggregated SCNT placentas were more similar to those of controls than to the profiles of SCNT placentas.

The data presented here indicate that the number of differentially deregulated genes in the SCNT placentas was decreased by 94.2% (85 → 5) in the aggregated SCNT placentas. These results are consistent with those of a direct comparison between gene expression in cumulus and ES cells of SCNT placentas, where there was a similar number of deregulated genes between both cell types [13]. Inappropriate reprogramming frequently occurs in somatic cell-cloned embryos [21], resulting in various deregulated gene expression patterns and epigenetic modifications in both the placenta and fetus [13]. Thus, the differences in gene expression were remarkably reduced by the aggregated SCNT method. The majority of aberrantly expressed genes were common to placentas cloned with ES or cumulus cells [13]. This indicated that placentomegaly in cloned mice is independent of the nuclear source of donor cells [6, 22, 23]. Therefore, aggregation of the tetraploid embryos utilized in this study have the key potential to reduce aberrant gene expression during the production of cloned mice, regardless of the nucleus source.

Many abnormalities in cloned animals suggest imprinting disruptions [24]. Placentomegaly was observed upon deregulation of imprinting genes such as *H19* [12], *Esx1* [10], and *Ipl* [11]. Although *H19* was shown to be one of the variable genes among cloned animals, its expression showed no variability in the presented microarray results, confirming previous results [13, 25]. It is also reported that the expression of insulin-like growth factor 2 receptor (*Igf2r*) was increased in placentomegaly [26]. In the present study, *Igf2r* expression was marginally reduced. Most of the identified imprinted genes showed a decreased expression. These results indicate that the decreased expression of those imprinted genes is caused by reduced expression of the normally active allele [25, 27]. However, only three genes *Slc22a18*, *Slc38a4*, and *Igfbp6* were expressed differentially between both placentas of SCNT and control placentas.

In the “biological process” category, the largest number of deregulated genes represented signal transduction (14%) proteins. In contrast, the largest number of genes in the molecular function category remained unclassified. Thus, it was very difficult to identify the specific causative genes of placentomegaly in SCNT placenta, but the condition seems to be caused by multiple-gene dysfunction. In the present study, we assessed differences in the expression of placenta-specific genes between SCNT and controls by qRT-PCR. These differences in expression were confirmed for eight genes. *Plac1*, a placenta-specific gene, is known to be expressed exclusively by the cells of the trophoblastic lineage in mice [28]. The other seven genes *Slc38a4*, *Rprml*, *Pla2g4f*, *Hsd17b7*, *Hmox1*, *Car2*, and *Pla2g4d* were specifically expressed in SCNT placentas. In the present study, *Plac1* is considered a candidate gene involved in placentomegaly in NT placentas, as reported previously [14]. Thus, these genes need to be systematically studied to resolve placentomegaly.

The altered expression of hundreds of genes in SCNT placentas may be related to the high mortality rate of cloned embryos [13]. According to Miki et al. [19], the extraembryonic lineages could be composed of tetraploid cells, the population of which was increased in full-term placental tissues. Specifically, tetraploid chimeras are considered the most outstanding result, since they enable the production of whole stem cell-derived mice offspring, whereas offspring could not be produced using the inner cell mass and pluripotent cells in chimeric rhesus monkeys [20, 29]. In the present study, our results are consistent with those of previous reports indicating that most clones show gene expression abnormalities resulting in subtle phenotype changes [30, 31], premature death [32], placental hyperplasia [19], or obesity [33].

These results are thought to be caused by aggregation of tetraploid embryos leading to the recovery of downregulated gene expression in the SCNT placentas.

## Conclusions

In summary, we present list of up- and down-regulated genes in the two types of SCNT and in vivo fertilization placentas. The expression of 206 (1.6%) of the 12,816 genes was found to be different by at least 2-fold between the SCNT placentas and controls. Further, 159 genes showed differential expression between the SCNT placentas and the aggregated SCNT placentas. However, gene expression profiles of the aggregated SCNT placentas were more similar to those of the controls than to those of the SCNT placentas. These results indicate that the aggregation SCNT technique using tetraploid embryos considerably decreased the number of deregulated genes by 94.2% (85 → 5) in the SCNT placentas. Therefore, aggregation with tetraploid embryos reduced abnormal gene expression in a genome-wide manner in the cloned placentas. Further studies will be needed to outline the molecular and functional mechanisms underlying abnormal expression of placenta-specific genes derived from tetraploid and cloned embryos.

## Additional files

Additional file 1: Table S1. List of primers used for real-time PCR. Genes from five SCNT placentas and three control placentas were analyzed. Twelve genes from different categories were chosen for qRT-PCR analyses. The gene for β-actin was used as the endogenous control. (PPTX 65 kb)

Additional file 2: Figure S1. Gene ontology of biological process. Gene ontology (GO) pie diagram of >2-fold differentially expressed genes between control and SCNT placentas. The upregulated or downregulated genes are categorized by the GO term “biological process.” (PPTX 152 kb)

Additional file 3: Figure S2. Gene ontology of molecular function. Gene ontology (GO) pie diagram of >2-fold differentially expressed genes between control and SCNT placentas. The upregulated or downregulated genes are categorized by the GO term “molecular function”. (PPTX 133 kb)

## Abbreviations

ES: Embryonic stem
GO: Gene ontology
LOS: Large offspring syndrome
qRT-PCR: Quantitative real-time PCR
SCNT: Somatic cell nuclear transfer
