# Supplementary material for: Abnormal gene expression in regular and aggregated somatic cell nuclear transfer placentas
Source: BMC Biotechnol. 2017 Mar 27;17:34. doi: 10.1186/s12896-017-0355-4 (PMC5368936; doi:10.1186/s12896-017-0355-4)
Supplement: Supplementary file 1 — List of primers used for real-time PCR. Genes from five SCNT placentas and three control placentas were analyzed. Twelve genes from different categories were chosen for qRT-PCR analyses. The gene for β-actin was used as the endogenous control. (PPTX 65 kb) [file 12896_2017_355_MOESM1_ESM.pptx]

## Slide 1
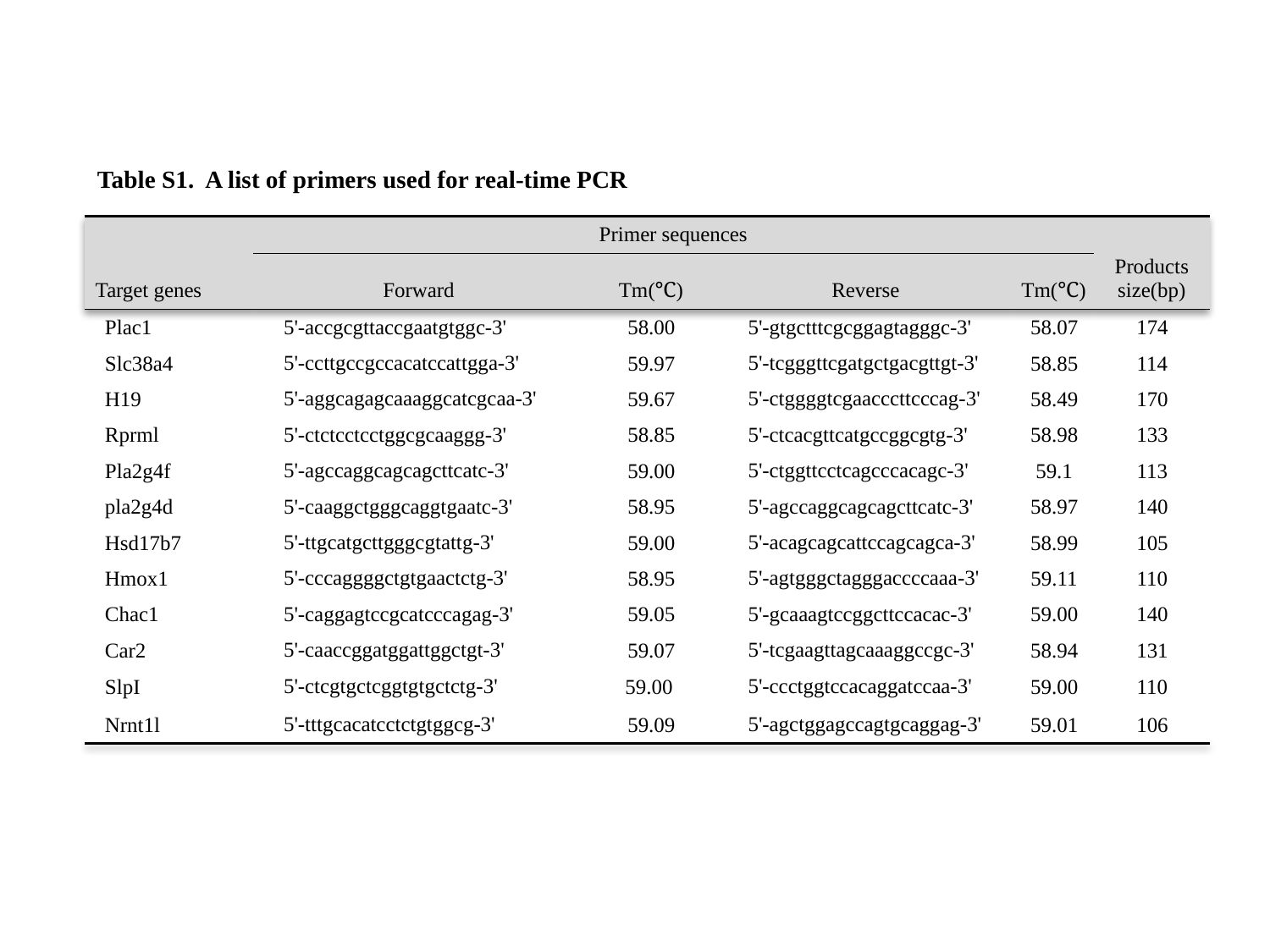

Table S1. A list of primers used for real-time PCR
| Target genes | Primer sequences | | | | Products size(bp) |
| --- | --- | --- | --- | --- | --- |
| | Forward | Tm(℃) | Reverse | Tm(℃) | |
| Plac1 | 5'-accgcgttaccgaatgtggc-3' | 58.00 | 5'-gtgctttcgcggagtagggc-3' | 58.07 | 174 |
| Slc38a4 | 5'-ccttgccgccacatccattgga-3' | 59.97 | 5'-tcgggttcgatgctgacgttgt-3' | 58.85 | 114 |
| H19 | 5'-aggcagagcaaaggcatcgcaa-3' | 59.67 | 5'-ctggggtcgaacccttcccag-3' | 58.49 | 170 |
| Rprml | 5'-ctctcctcctggcgcaaggg-3' | 58.85 | 5'-ctcacgttcatgccggcgtg-3' | 58.98 | 133 |
| Pla2g4f | 5'-agccaggcagcagcttcatc-3' | 59.00 | 5'-ctggttcctcagcccacagc-3' | 59.1 | 113 |
| pla2g4d | 5'-caaggctgggcaggtgaatc-3' | 58.95 | 5'-agccaggcagcagcttcatc-3' | 58.97 | 140 |
| Hsd17b7 | 5'-ttgcatgcttgggcgtattg-3' | 59.00 | 5'-acagcagcattccagcagca-3' | 58.99 | 105 |
| Hmox1 | 5'-cccaggggctgtgaactctg-3' | 58.95 | 5'-agtgggctagggaccccaaa-3' | 59.11 | 110 |
| Chac1 | 5'-caggagtccgcatcccagag-3' | 59.05 | 5'-gcaaagtccggcttccacac-3' | 59.00 | 140 |
| Car2 | 5'-caaccggatggattggctgt-3' | 59.07 | 5'-tcgaagttagcaaaggccgc-3' | 58.94 | 131 |
| SlpI | 5'-ctcgtgctcggtgtgctctg-3' | 59.00 | 5'-ccctggtccacaggatccaa-3' | 59.00 | 110 |
| Nrnt1l | 5'-tttgcacatcctctgtggcg-3' | 59.09 | 5'-agctggagccagtgcaggag-3' | 59.01 | 106 |
