# Supplementary figures and images for: Abnormal gene expression in regular and aggregated somatic cell nuclear transfer placentas
Source: BMC Biotechnol. 2017 Mar 27;17:34. doi: 10.1186/s12896-017-0355-4 (PMC5368936; doi:10.1186/s12896-017-0355-4)

## Slide 1
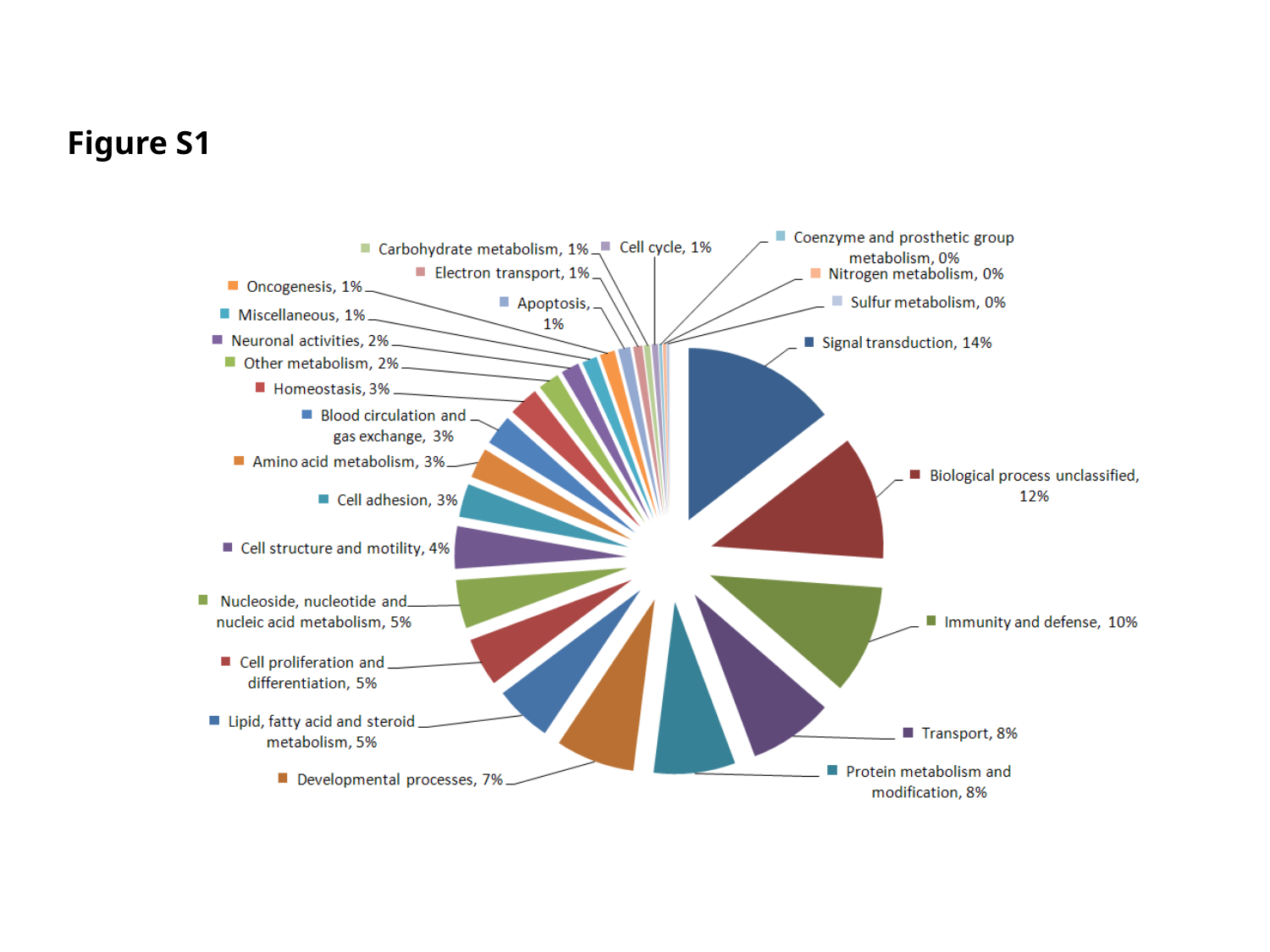

Figure S1

Supplement: Supplementary file 2 — Gene ontology of biological process. Gene ontology (GO) pie diagram of >2-fold differentially expressed genes between control and SCNT placentas. The upregulated or downregulated genes are categorized by the GO term “biological process.” (PPTX 152 kb) [file 12896_2017_355_MOESM2_ESM.pptx]

## Slide 1
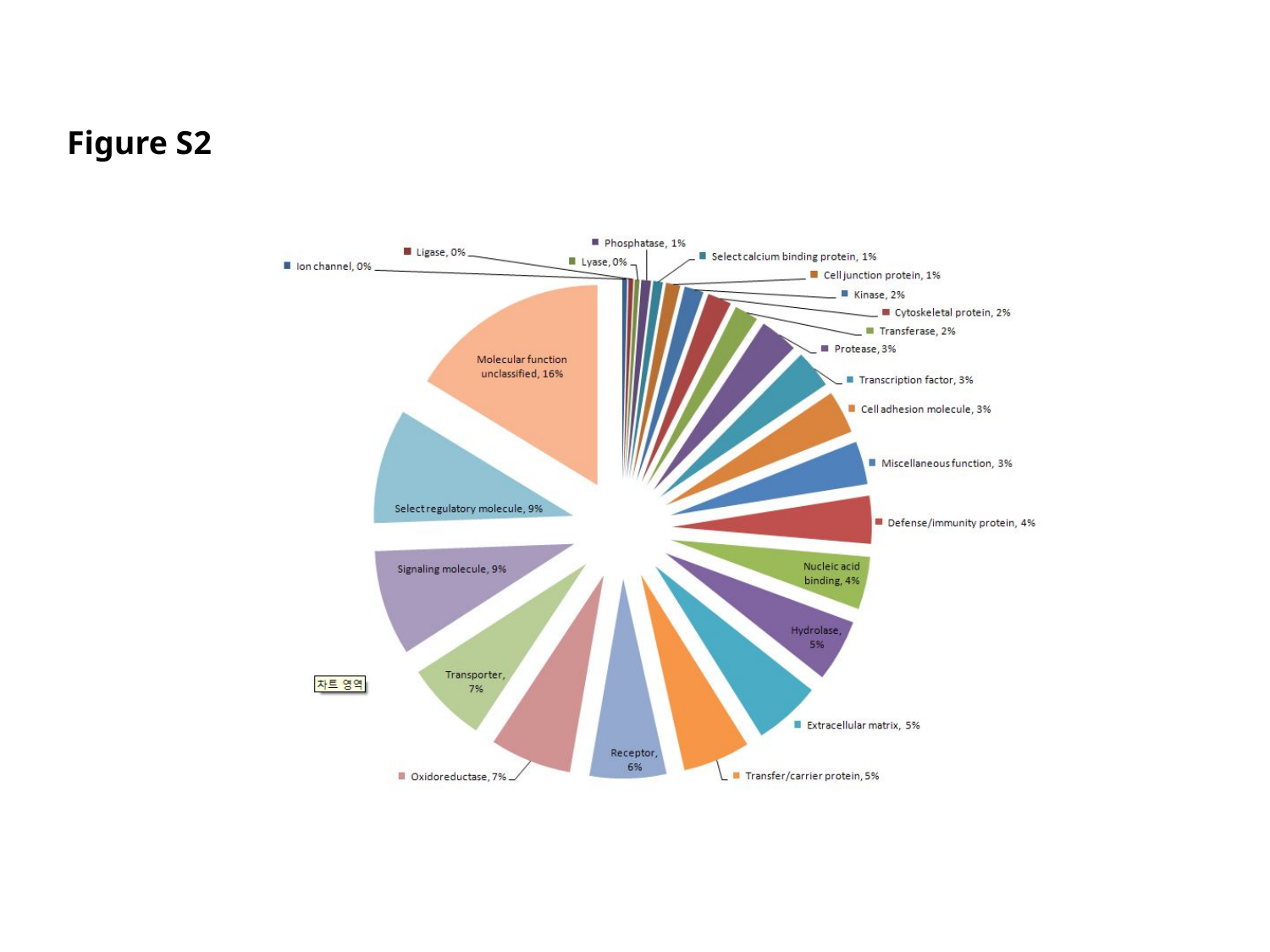

Figure S2

Supplement: Supplementary file 3 — Gene ontology of molecular function. Gene ontology (GO) pie diagram of >2-fold differentially expressed genes between control and SCNT placentas. The upregulated or downregulated genes are categorized by the GO term “molecular function”. (PPTX 133 kb) [file 12896_2017_355_MOESM3_ESM.pptx]
